# Evolution Mechanism of Multi-Precipitation Regulates Mechanical Properties and High-Temperature Strength in Medium-Alloy Structural Steel

**DOI:** 10.3390/ma18040848

**Published:** 2025-02-15

**Authors:** Junjie Sheng, Yahui Deng, Xin Cao, Yangxin Wang, Chundong Hu, Han Dong

**Affiliations:** 1 School of Materials Science and Engineering, Shanghai University, Shanghai 200444, China; sjj2000@shu.edu.cn (J.S.); yahui@shu.edu.cn (Y.D.); donghan@shu.edu.cn (H.D.); 2 Zhejiang Institute of Advanced Materials, Shanghai University, Jiaxing 314100, China; caoxin961113@163.com

**Keywords:** carbides, evolution mechanism, mechanical properties, high-temperature strength

## Abstract

Precipitation strengthening is one of the fundamental factors occurring at high temperatures in medium-alloy structural steels, which offer greater durability under service conditions. This research employed transmission electron microscopy (TEM) via carbon replicas combined with scanning electron microscopy (SEM) to analyze carbide evolution and its influence on both mechanical properties and high-temperature strength. During the tempering process, ε-carbides precipitate at 200 °C and subsequently transform into M_3_C at 400 °C and coarser M_7_C_3_ at 600 °C. Coarser carbides (M_7_C_3_ and M_3_C) and metastable carbides (ε-carbides) are not sufficient to make steel strong at high temperatures. Moreover, nucleating and growing at interfaces, rod-shaped M_3_C diminishes the toughness of the steel. Under tempering at 600 °C, a substantial amount of nanoscale M_2_C carbides precipitate. This improvement not only elevate the material’s toughness but also leads to an enhancement of yield strength (from 1237 ± 12 MPa to 1340 ± 8 MPa) along with a rise in high-temperature strength (from 388 ± 8 MPa to 421 ± 4 MPa). Combined with high toughness, nanoscale M_2_C with high thermal stability promoted both yield strength at room temperature and high-temperature strength. The type and size of carbides serve as key determinants for yield strength while being decisive parameters for high-temperature strength.

## 1. Introduction

Precipitates serve as essential microstructural components that determine the performance of medium-alloy steels [1,2]. Due to superior performance characteristics, Ci-Ni-Mo-V steels are commonly utilized for structural and industrial applications [3,4,5]. Combined with mechanical load, a significant amount of heat and repeated thermal cycling are essential causes of failure [6,7]. Studies have revealed that high-temperature strength and mechanical properties at room temperature are critical parameters affecting the service life of medium-alloy steels [8,9].

The mechanical strength of metallic substances is fundamentally governed by the impediments to dislocation motion within their structure, including grain boundaries, solute atoms, precipitates and dislocations [8,10,11]. Under high-temperature conditions, the reinforcement capability of grain boundaries becomes weakened, and the specific mechanisms responsible for this reduction are as follows: (1) The reduction in small-angle grain boundaries composed of dislocations [12,13]. (2) At high temperatures, the sliding of grain boundaries is significantly enhanced, which substantially reduces strength [14]. Carbides can strongly hinder the slip and climb of dislocations, enhancing high-temperature strength effectively. When tempering is conducted at relatively low temperatures, carbon atoms exhibit sufficient mobility, whereas substitutional alloying elements demonstrate insufficient diffusivity to induce notable alterations in the martensitic structure [15]. Consequently, below a 250 °C tempering temperature, mechanical characteristics are primarily governed by carbon-diffusion-induced transition carbides, resulting in superior strength combined with moderate toughness under ambient conditions [16]. When the temperature is raised above 300 °C, transition metastable carbides are replaced by more stable phases such as cementite [17]. Higher tempering temperatures (above 500 °C) allow other alloy elements to be mobile, transform into other precipitates and influence structural changes [18,19].

K. Kaneko [20] suggests that only Fe_3_C or M_3_C form at 300–500 °C. By elevating the tempering temperature above 600 °C, the evolution of carbides demonstrates varying transformation pathways: M_3_C → M_2_C → M_7_C_3_ + M_6_C → M_23_C_6_ [21], M_3_C + M_2_C + MC → M_7_C_3_ + M_2_C +MC → M_3_C + M_2_C + MC + M_7_C_3_ + M_23_C_6_ [22], M_3_C →M_2_C → M_7_C_3_ + M_23_C_6_ → M_23_C_6_ + M_6_C [23]. These results, above, demonstrate an inherent interdependence between precipitate formation and elemental redistribution throughout the tempering process. Current research predominantly concentrates on optimizing high-temperature tempering parameters to enhance elevated-temperature strength properties [8]. Nevertheless, the current understanding of how tempering temperature governs concurrent microstructural evolution and carbide behavior, subsequently impacting both room-temperature and high-temperature strength profiles in steels, remains incomplete.

In the present work, we developed a novel Cr-Ni-Mo-V steel. Through controlled tempering temperature of the steel, this work elucidates the temperature-dependent effects of the microstructure and carbides on mechanical properties and high-temperature strength. The aim is to delve into the high-temperature strengthening mechanisms of microstructural and carbide evolution, based on the foundation of controlling basic mechanical properties.

## 2. Materials and Methods

In this work, a novel Cr-Ni-Mo-V steel, with a nominal composition of Fe-0.33C-2.4Cr–3.1Ni–2.2Mo-0.8V-0.3Co (wt.%) was employed. The A_c1_, A_c3_, M_s_ and M_f_ temperatures of the steel, determined by dilatometry, were 736 °C, 864 °C, 337 °C and 183 °C, respectively. It was specifically designed for applications involving high-temperature and high-pressure industrial production. The alloy fabrication process integrated vacuum induction melting (VIM) with subsequent electroslag remelting (ESR) treatments. The material underwent extended diffusion treatment at 1250 °C (30 h duration) prior to controlled furnace cooling, ultimately achieving 230 mm diameter and 9000 mm length ingot formation. Prior to heat treatment, all specimens intended for subsequent testing were extracted from the mid-radius position of the steel ingot. After standard austenitization (980 °C × 1 h) and oil quenching operations, the specimens were subjected to 2 h tempering treatments at five discrete temperatures (200 °C, 400 °C, 500 °C, 600 °C, 700 °C).

**Figure 1:**
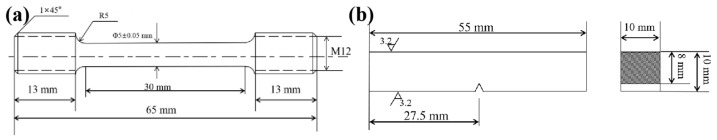
Schematic illustration of mechanical test specimens: (**a**) Tensile bar and (**b**) Charpy V-notch impact sample.

Following GB/T228.1-2010 [24] and GB/T228.2-2015 [25] standards, uniaxial tensile testing was performed on an Instron 5982 system (Instron, Norwood, MA, USA) under two thermal conditions: ambient temperature and 700 °C, with a controlled strain rate of 1 × 10^−3^ s^−1^ at room temperature and 700 °C. Specimens underwent 15 min thermal stabilization at 700 °C prior to elevated-temperature testing to ensure thermal equilibrium. The tensile specimens were all taken parallel to the axial direction for testing. Considering the service conditions, the impact test specimens were taken perpendicular to the axial direction. The temperature of −40 °C is of particular interest for engineering steel or structural steel used in extreme environments. Low-temperature impact tests (−40 °C) were carried out with the NI750 Charpy testing apparatus (Steel Research National Engineering Technology Co., Ltd., Beijing, China) following ASTM E23 standards [26]. The fracture mechanisms were investigated through scanning electron microscopy (SEM) observations of impact-tested samples’ failure surfaces. Figure 1 shows specimen dimensions subjected to impact and tensile tests.

Microstructural characterization of differentially tempered specimens was performed via SEM (FEI Apreo 2S HiVac, Thermo Fisher Scientific, Waltham, MA, USA) and electron backscatter diffraction (EBSD) to elucidate precipitation behavior and strengthening mechanisms in steel. Specimen preparation involved sequential grinding/polishing followed by 8 vol.% nital etching for SEM analysis and vibratory polishing for EBSD examination. The EBSD characterization was acquired under optimized conditions: 20 kV accelerating voltage and 0.12 μm scan step resolution. The data were post-processed by the AZtecCrystal software (Version 2.1), where the geometrically necessary dislocation (GND) and phase distribution were obtained.

Projection specimens were fabricated through a combined electrolytic twin-jet thinning and carbon replica technique. Electrolytic twin-jet thinning was conducted using a TJ-100SE type device (CUI Inc., Portland, OR, USA) under cryogenic conditions (−30 °C) with a 30 kV applied voltage. Metallographically prepared samples served as base materials for carbon extraction replica fabrication. Initial chemical treatment involved 4 vol.% nital etching prior to vapor deposition of 15–30 nm thick carbon films. Post-deposition processing included mechanical scoring (1.5 mm^2^ grids) and secondary etching with 10 vol.% nital for matrix dissolution, followed by controlled replica flotation. Ultimate replica transfer was achieved using copper support grids with filter paper-assisted drying. Microstructural and replica characterization was performed using a JEM-F200 (JEOL Ltd., Tokyo, Japan) field emission transmission electron microscope (TEM).

To better demonstrate the tests conducted on specimens in different conditions, the tests performed on specimen at various tempering temperatures are shown in Table 1.

## 3. Results and Discussion

### 3.1. Mechanical Properties and High-Temperature Strength

**Figure 2:**
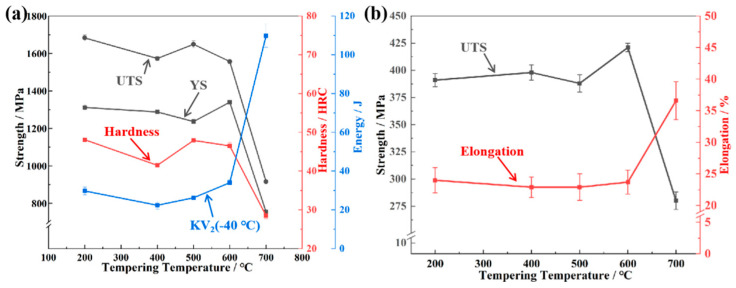
(**a**) The mechanical properties, (**b**) UTS and elongation at 700 °C under different heat treatment conditions.

Figure 2a illustrates the mechanical properties of the steel subjected to the following sequential heat treatments: an initial 980 °C/1 h austenitization with oil quenching, followed by 2 h tempering across different temperature (UTS = ultimate tensile strength; YS = yield strength). The results indicate that the mechanical properties of this steel exhibit varying behaviors at different tempering temperatures. Exhibiting characteristic secondary hardening behavior, the steel’s tensile strength displays a dip-and-rise evolution with tempering temperature elevation. The hardness exhibits a similar dependence on tempering temperature to that of tensile strength, reaching a secondary hardening peak of 47.9 ± 0.2 HRC at 500 °C. In more detail, a tensile strength minimum (1573 ± 10 MPa) occurs at 400 °C tempering. Subsequently, the tensile strength reaches its peak of secondary hardening at 500 °C, with a value of 1649 ± 19 MPa. On the other hand, the impact absorption energy (−40 °C) is its minimum of 22.4 ± 2.1 J at 400 °C, and reaches a peak of 34.0 ± 1.4 J at 600 °C. Distinctively, the steel exhibits a significant impact energy value of 109.8 ± 6 J upon tempering at 700 °C, coincident with a sharp decrease in strength. A notable inverse relationship emerges between tensile and yield strengths when tempering temperature increases from 400 °C to 500 °C: while tensile strength rises, yield strength declines from 1288 ± 11 MPa to 1237 ± 12 MPa. This inverse correlation persists throughout the 400–600 °C tempering regime, with tensile strength showing positive temperature dependence and yield strength exhibiting negative correlation.

Figure 2b depicts the mechanical response (ultimate tensile strength and elongation) of the steel to tempering temperature under uniaxial tensile testing conditions at 700 °C. When the tempering temperature is between 200 and 500 °C, the high-temperature strength changes little and is relatively close. Subsequently, as the tempering temperature rises, the high-temperature strength exhibits a trend that is consistent with the yield strength at room temperature in response to changes in tempering temperature: The strength initially increases to a peak value of 421 ± 4 MPa at 600 °C, and then decreases. Beyond 600 °C, the UTS rapidly diminishes with increasing tempering temperature, dropping to 280 ± 8 MPa at 700 °C. Furthermore, at a tensile testing temperature of 700 °C, the higher the strength of the experimental steel, the lower the elongation, which aligns with the trade-off relationship between strength and ductility. With the variation in tempering temperature, the yield strength undergoes changes. Intriguingly, it affects the high-temperature strength in a similar pattern. This correlation may be attributed to the different precipitates and microstructural evolution within the material.

### 3.2. Microstructure Evolution

**Figure 3:**
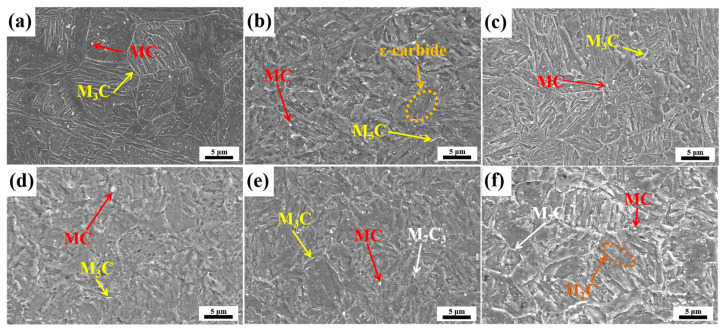
The microstructures of the materials (**a**) as-quenched and tempered at (**b**) 200 °C, (**c**) 400 °C, (**d**) 500 °C, (**e**) 600 °C, (**f**) 700 °C.

**Figure A1:**
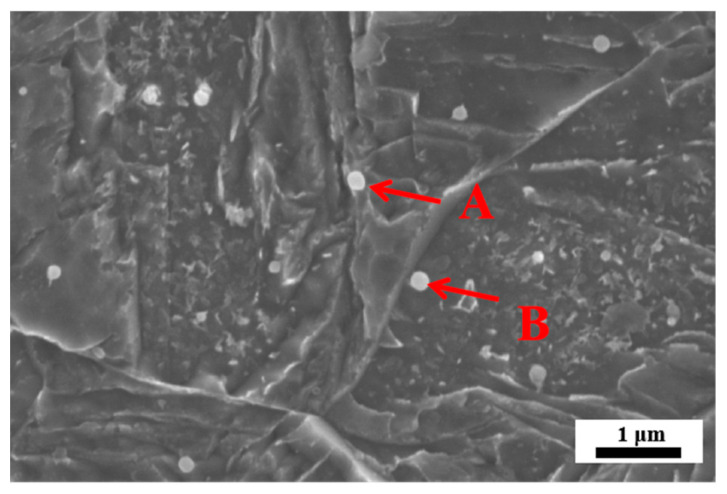
The SEM microstructure of the as-quenched steel and the positions scanned by EDS point scanning.

Figure 3a shows the microstructure of the as-quenched materials, which contain fully typical lath-like martensite. The as-quenched martensitic microstructure is adorned with quasi-spherical fine carbides and strip-like carbides distributed along interfaces of martensite laths and prior austenite grain boundaries. These carbides remain undissolved in the matrix during the quenching process and are thus retained within the microstructure. Subsequently, as shown in Figure 3b–e, during the tempering process, these carbides are preserved. The energy-dispersive X-ray spectroscopy (EDS) results (Figure A1) reveal an atomic mass ratio of 1.25:1 between vanadium (V) and carbon (C) elements in the globular carbides, which confirms that these carbides are of the MC (M represents V) type. Figure 3b–f demonstrate the microstructures of the materials at different tempering temperatures. Strip-like carbides maintain their lath-boundary distribution pattern. Moreover, elevated tempering temperatures do not induce measurable coarsening in prior austenite grains.

Needle-like carbides can be observed distributed along the martensite laths at a tempering temperature of 200 °C, as shown in Figure 3b. Elevated tempering temperatures induce the dissolution of the needle-like carbides. Martensite recovery and carbide precipitation constitute coupled processes throughout tempering [27]. When the tempering temperature reaches 500 °C, partial boundaries of martensite blocks and packets begin to blur, C begins to precipitate out of the martensite, and martensite laths with high densities of dislocations undergo recovery. Upon the further elevation of temperature, the steel remains in a martensitic microstructure. Figure 3e demonstrates that 700 °C tempering triggers dual microstructural events: enhanced matrix recovery and accelerated carbide precipitation and growth [28,29]. A dramatic drop in strength indicates a loss of strengthening mechanisms in the microstructure following 700 °C thermal exposure.

**Figure 4:**
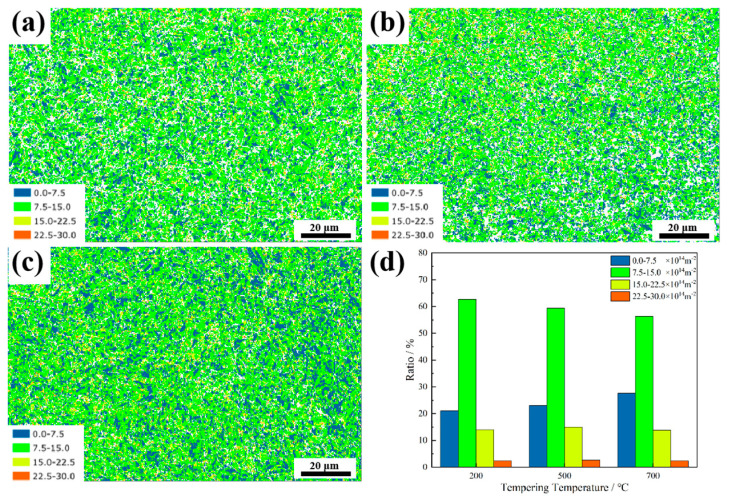
The variation in GND density of the steels tempered at (**a**) 200 °C, (**b**) 500 °C, (**c**) 700 °C. (**d**) The varying content ratios of different GND densities with changes in tempering temperature.

Figure 4a–c illustrate the variation in the geometrically necessary dislocation (GND) density of the specimens tempered at different temperatures. The proportion of different GNDs at various tempering temperatures is shown in Figure 4d. This steel has a high density of GND. As the temperature increases, the proportion of low-GND areas (0.0–7.5 × 10^14^ m^−2^) (blue areas in Figure 4a–c) gradually increases, while the proportion of GND in the range of 7.5–15 × 10^14^ m^−2^ (red areas in Figure 4a–c) gradually decreases. This is characteristic of the recovery process at elevated tempering temperatures, where high-energy dislocation configurations are less stable at higher temperatures and tend to reduce. Moreover, the reduction in dislocations contributes to a loss of strength, so when the temperature reaches 700 °C, the decrease in strength is more considerable. Compared to 200 °C, the GND at 500 °C is slightly reduced, as shown in Figure 4a,b. However, the strength of the steel still remains at a relatively high level. This suggests that, in addition to the influence of GNDs, other factors such as microstructure and carbides prevent the strength from decreasing along with the reduction in dislocations.

### 3.3. Carbide Evolution and Strengthening Mechanism

Alongside matrix restoration, Figure 3 reveals dynamic changes in precipitate morphology and distribution. These could have a substantial influence on the strength, toughness and high-temperature strength. Steels typically contain multiple precipitate variants such as ε-carbide, MC, M_2_C, M_3_C, M_6_C, M_7_C_3_ and M_23_C_6_ carbide, Laves phase and so on [27,30]. Phase identification was accomplished through the systematic analysis of the characteristic diffractions of these precipitates.

**Figure 5:**
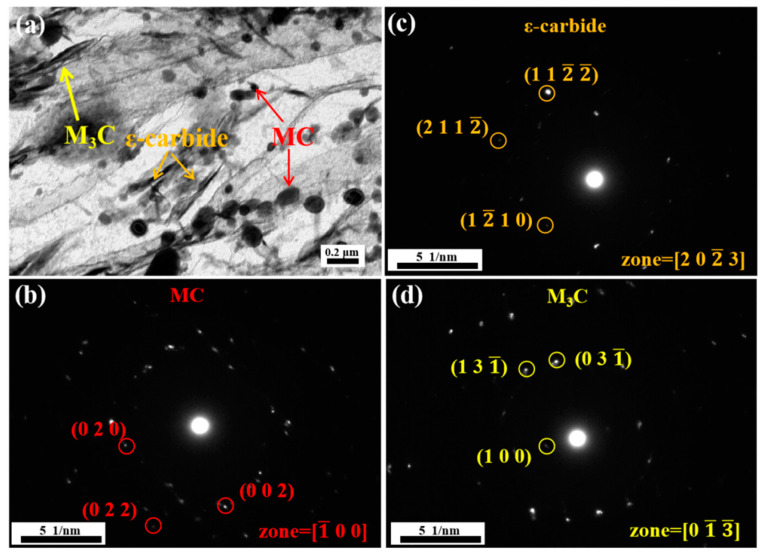
TEM images of replica of steel tempered at 200 °C: (**a**) bright field image, (**b**–**d**) diffraction patterns of ε-carbide, MC and M_3_C, respectively.

**Figure A2:**
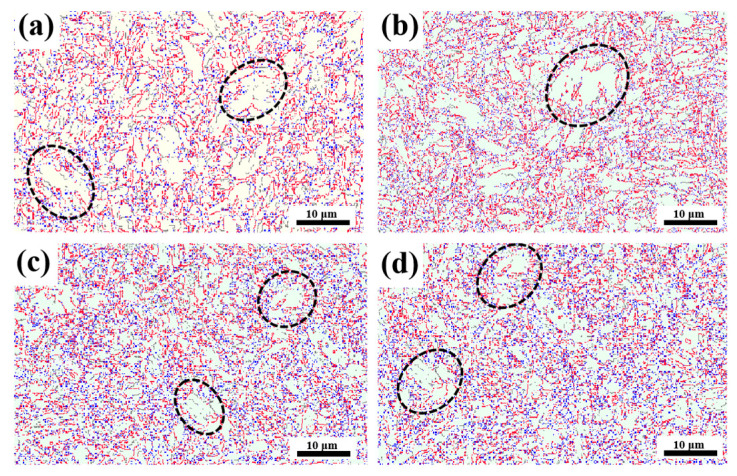
The grain boundary and phase maps of steels tempered at (**a**) 200 °C, (**b**) 400 °C, (**c**) 500 °C, (**d**) 600 °C.

Figure 5a shows the TEM micrograph of the replica obtained from the material tempered at 200 °C. We observed the presence of needle-like ε-carbides, M_3_C and spherical MC carbides in the replica sample. There are a considerable number of ε-carbides (orange arrow) with lengths ranging from 229 nm to 281 nm, a certain quantity of M_3_C (yellow arrow) carbides with lengths between 278 nm and 386 nm, and MC (red arrow) carbides with equivalent diameters spanning from 185 nm to 331 nm. The MC-type carbides are not formed at 200 °C, they are the result of the undissolved carbides left over from the quenching process, as shown in Figure 3a. Additionally, the typical formation temperature for M_3_C carbides is above 300 °C [31,32]. ε-carbides are the predominant precipitates at 200 °C, primarily composed of Fe and C atoms, with the composition Fe_(2-3)_C [33]. This type of carbide is predominantly precipitated between the martensitic laths and in a semi-coherent state with the matrix. During the deformation process, sub-micron ε-carbides play a strengthening role through their interaction with dislocations, which makes the steel strong at 200 °C. However, the metastable ε-carbides exhibit low thermal stability at high temperatures and are prone to transforming into larger M_3_C under tensile testing at 700 °C. Additionally, M_3_C carbides are widely present at the interfaces and are more likely to nucleate and grow on these interfaces, as shown in Figure A2. The larger carbide (M_3_C) has an inferior interaction with dislocations at high temperatures; therefore, its high-temperature strength is relatively low.

**Figure 6:**
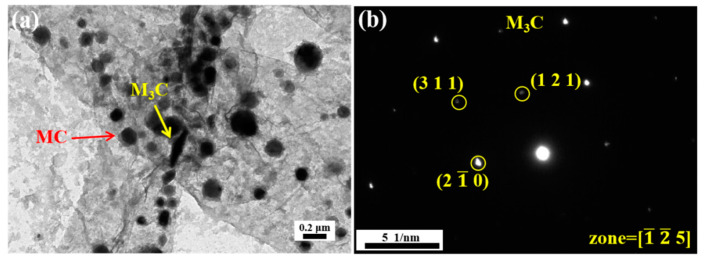
TEM images of replica of steel tempered at 400 °C: (**a**) bright field image, (**b**) diffraction pattern of M_3_C.

Figure 6 shows that only MC and M_3_C carbides were detected, and the diffraction pattern demonstrates the rod-like carbides were M_3_C. The MC carbides measure 140–168 nm and M_3_C carbides range from 189 to 476 nm in size. Compared to 200 °C, after tempering at 400 °C, the ε-carbide disappears. Moreover, the size and quantity of MC carbides show no significant difference. When elevating the tempering temperature to 400 °C, the metastable ε-carbide in the general tempered martensite transforms to M_3_C [34]. Coarse M_3_C carbides demonstrate limited strengthening capacity, while their precipitation/growth correlates with C depletion from the martensitic matrix. The synergistic effect of matrix carbon loss and insufficient carbide strengthening leads to an overall strength reduction.

**Figure 7:**
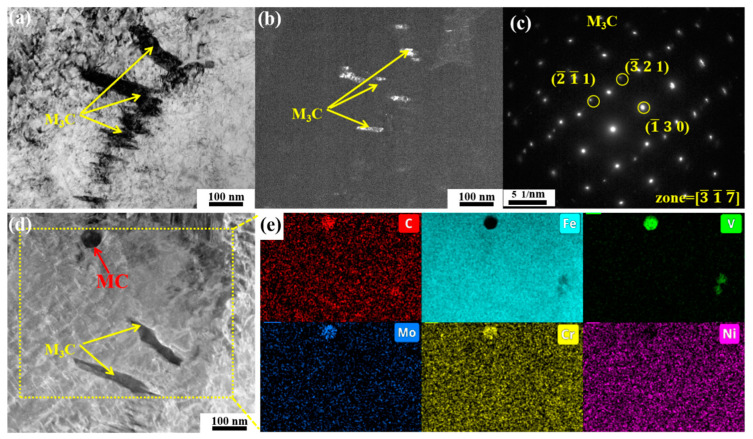
TEM images of specimen tempered at 400 °C: (**a**,**d**) bright field, (**b**) dark field of M_3_C, (**c**) diffraction pattern of M_3_C, (**e**) EDS mapping of yellow-dotted region in (**d**).

The bright field micrograph of the 400 °C tempered specimen is presented in Figure 7a, while Figure 7b illustrates the corresponding dark field image of the M_3_C carbides. The majority of the M_3_C carbides retain their relatively coarse state after quenching, and several relatively fine M_3_C carbides begin to precipitate. As demonstrated in Figure 7d,e through EDS analysis, the spheroidal carbides correspond to MC carbides, whereas the rod-shaped M3C phase predominantly contains C and Fe elements. Therefore, the M_3_C in this state is identified as Fe_3_C.

**Figure 8:**
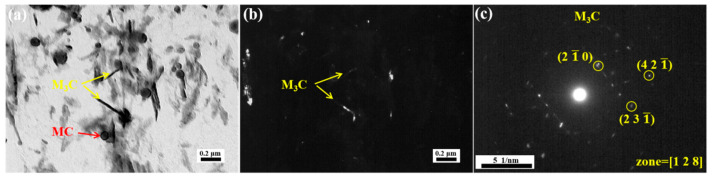
TEM images of replica of steel tempered at 500 °C: (**a**) bright field image, (**b**) dark field image of M3C, (**c**) diffraction pattern of M_3_C.

**Figure A3:**
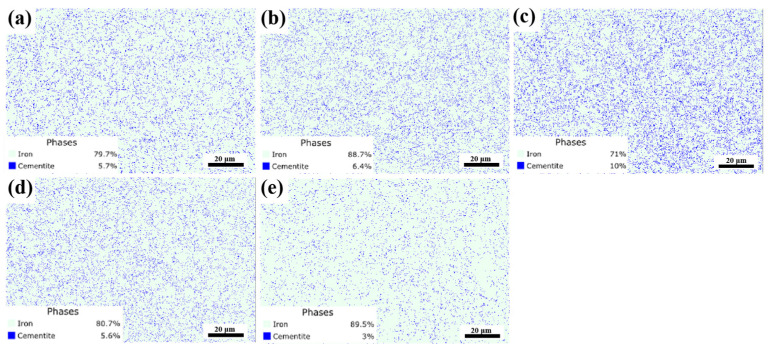
The phase distribution map obtained through the EBSD of steels tempered at (**a**) 200 °C, (**b**) 400 °C, (**c**) 500 °C, (**d**) 600 °C, (**e**) 700 °C.

Figure 8a demonstrates that MC carbides and M_3_C carbides are present at 500 °C. Compared to the M_3_C carbides at 400 °C, there are finer M_3_C carbides at 500 °C, with the average length decreased from 333 ± 46 nm to 255 ± 54 nm. In addition, a marked increase in the number of M_3_C carbides is observed, as shown in Figure 8b. This indicates a substantial precipitation of M_3_C at 500 °C, which is consistent with Figure A3. The freshly precipitated slender M_3_C can form an Orowan mechanism with dislocations, providing a certain strengthening effect. However, as discussed above, the contribution of M_3_C to high-temperature strength is limited [29].

**Figure 9:**
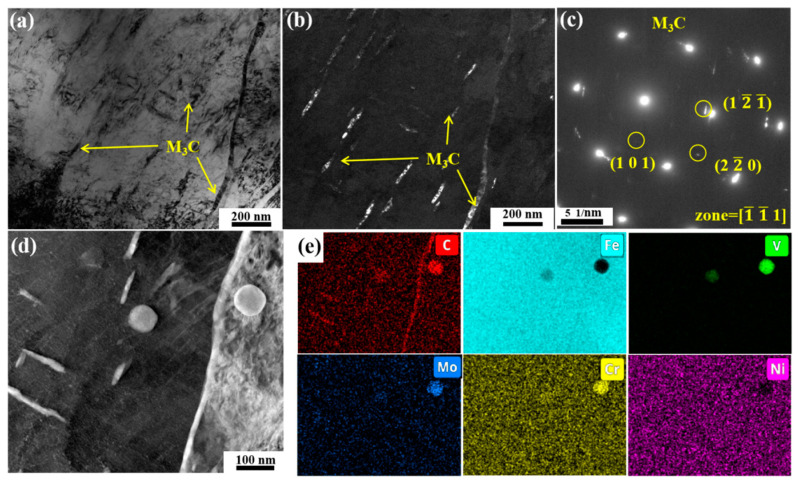
TEM images of specimen tempered at 500 °C: (**a**,**d**) bright field, (**b**) dark field of M_3_C, (**c**) diffraction pattern of M_3_C, (**e**) EDS mapping of (**d**).

Figure 9a,b present the bright field micrograph of the 500 °C tempered specimen and the corresponding dark field image of its M_3_C phase, respectively. Consistent with the observations in Figure 8a, Figure 9b reveals abundant nanoscale M_3_C precipitates formed during 500 °C tempering. Moreover, it can be observed that elongated M_3_C forms on the grain boundaries, as evidenced in Figure 9b,d. As depicted in Figure 9d,e, the spherical MC carbides show no significant changes at 500 °C, with both intragranular and intergranular M_3_C precipitates being characterized as Fe_3_C. The dispersed distribution of Fe_3_C leads to an increase in strength. However, some Fe_3_C present at the grain boundaries may have a detrimental effect on toughness.

**Figure 10:**
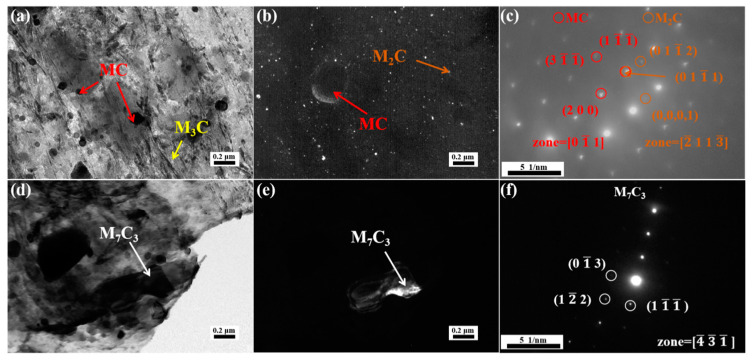
TEM images of replica of steel tempered at 600 °C: (**a**) bright field image, (**b**) dark field image of M_2_C, (**c**) diffraction patterns of MC and M_2_C; (**d**,**e**) bright and dark field images of M_7_C_3_, (**f**) diffraction pattern of M_7_C_3_.

Figure 10a,b indicate that the amount of M_3_C decreases when tempered at 600 °C. Additionally, rod-shaped and lump-shaped M_7_C_3_ (white arrow) carbides emerge in certain regions, as shown in Figure 10a,d. The average equivalent diameter of M_7_C_3_ is about 123 ± 58 nm. These M_7_C_3_ carbides may have originated from the transformation of M_3_C carbides [22]. Concurrently, although the average size of the MC carbides has not changed significantly, there are occasional instances of larger MC carbides with equivalent diameters reaching up to over 170 nm. Distinctively, a large number of granular carbides at the nanoscale precipitate, with an average equivalent diameter of approximately 14 ± 2 nm. The carbides can be identified as M_2_C (brown arrow) with a hexagonal close-packed (HCP) structure in Figure 10c. Fine M_2_C carbides with equivalent diameters ranging from 13 nm to 16 nm begin to precipitate extensively, as shown in Figure 10b. As the nucleation of M_2_C carbides generally depends on dislocations [35], the matrix exhibiting elevated dislocation densities induces the uniform and fine precipitations of M_2_C carbides. Therefore, under tempering conditions at 600 °C, a multitude of nanoscale M_2_C carbides precipitate. The precipitated M_2_C interacts with dislocations through a by-pass mechanism at this temperature [36]. The Orowan dislocation loop model has been predominantly applied in prior investigations to quantify strengthening contribution of M_2_C carbide precipitation, with the equations describing this strengthening mechanism detailed in Refs. [36,37]:

(1) $$∆\sigma_{M_{2}C}=MY\frac{2Kb}{w_{L}R}\ln(\frac{2w_{D}R}{b})\sqrt{\frac{\ln\left(\frac{2w_{D}R}{b}\right)}{\ln\left(\frac{w_{L}R}{b}\right)}}$$

where $K={G(1/0.7)}^{0.5}/4\pi$, $w_{L}={\left(\pi w_{q}/\varphi_{M_{2}C}\right)}^{0.5}-2w_{r}$ and $1/w_{D}=1/w_{L}+1/w_{R}$. Therefore, minute and numerous M_2_C particles can produce a higher level of precipitation strengthening [36,37,38]. This corresponds to a significant increase in the yield strength. Furthermore, the thermal stability of M_2_C is much greater than that of the ε-carbides and M_3_C, which allows higher high-temperature strength. Therefore, it can provide higher high-temperature strength [22].

**Figure 11:**
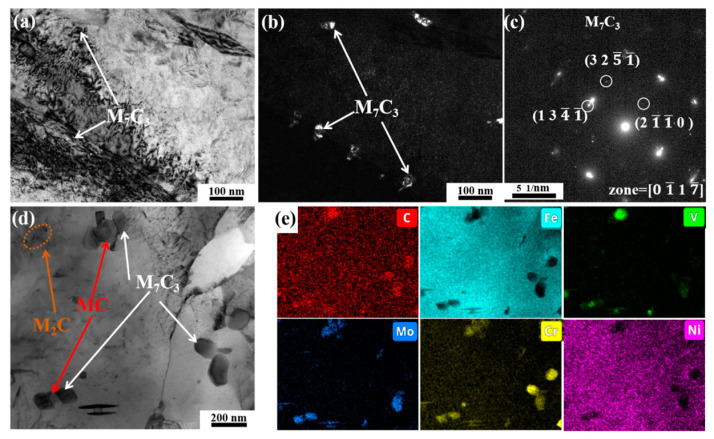
TEM images of the specimen tempered at 600 °C: (**a**,**d**) bright field, (**b**) dark field of M_7_C_3_, (**c**) diffraction pattern of M_3_C, (**e**) EDS mapping of (**d**).

**Figure A4:**
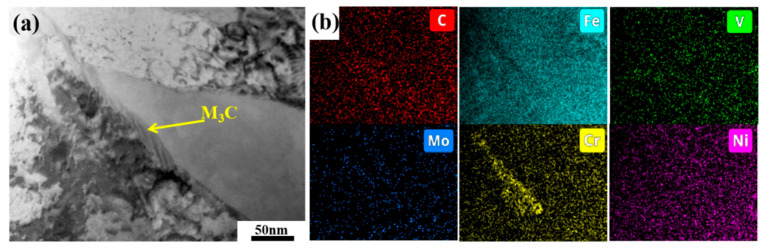
TEM image and EDS mapping of M_3_C in specimen tempered at 600 °C: (**a**) bright field image, (**b**) EDS mapping.

The microstructural evolution captured in Figure 11a–c reveals the initial precipitation of M_7_C_3_ carbides at 600 °C, showing fine correspondence with the phase transformation behavior documented in Figure 10e. Moreover, the lump-like M_7_C_3_ is a Cr- and Mo-rich phase, as shown in Figure 11d,e. Furthermore, fine acicular M_2_C rich in Mo and Cr can also be observed. Interestingly, dumbbell-like composite carbides can be observed in Figure 11d. According to Figure 11e, the MC carbides are located in the middle of the dumbbell-shaped carbides, while the M_7_C_3_ carbide are located on both sides of the MC carbides. In addition, although M_3_C significantly decreases at 600 °C, a small amount of M_3_C still exists. At this temperature, M_3_C transforms from Fe_3_C to alloyed cementite, as shown in Figure A4.

**Figure 12:**
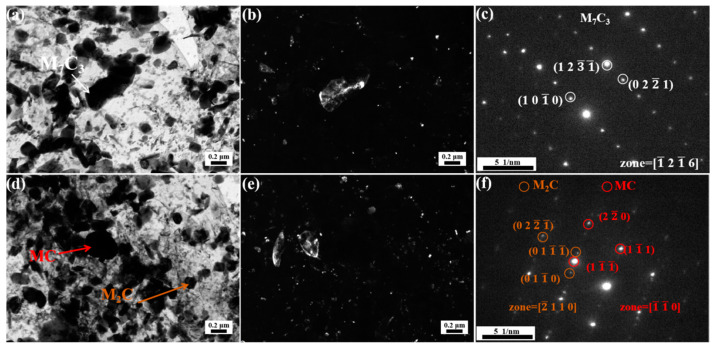
TEM images of replica of steel tempered at 700 °C: (**a**,**d**) bright field image, (**b**,**e**) dark field images of M_7_C_3_ and M_2_C, respectively, (**c**) diffraction pattern of M_7_C_3_, (**f**) diffraction patterns of MC and M_2_C.

Figure 12 shows that a substantial amount of large-sized carbides precipitate while M_3_C carbides disappear, at 700 °C. The M_3_C may have transformed completely into M_7_C_3_. Moreover, all carbides coarsen, including the MC carbides that are not completely dissolved in the quenched state. The average equivalent diameters of MC, M_2_C and M_7_C_3_ grow up to 201 ± 98 nm, 36 ± 6 nm and 146 ± 57 nm, respectively. In particular, there is a certain amount of large-sized M_7_C_3_ carbides which are larger than 650 nm. Although the precipitation of large-sized carbides contributes to precipitation strengthening, the extensive precipitation and coarsening of these carbides can lead to a decrease in the solid solubility of the alloying elements and carbon. This diminished solid solubility directly induces a substantial attenuation of solid-solution strengthening effects. Consequently, pronounced degradation in both yield strength and high-temperature mechanical performance occurs.

**Figure 13:**
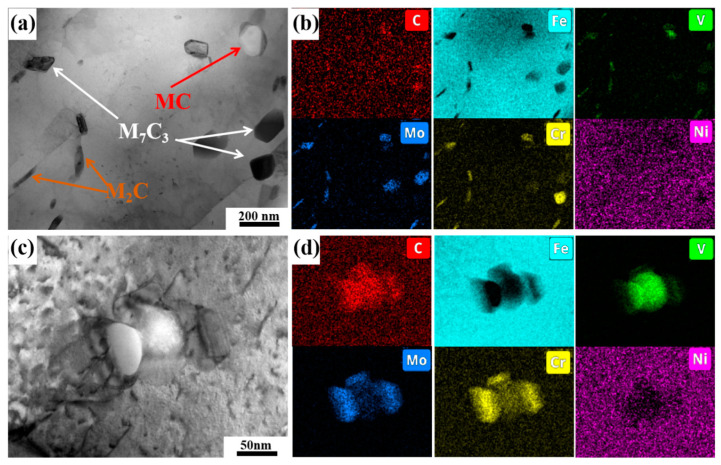
TEM images of specimen tempered at 700 °C: (**a**,**c**) bright field, (**b**,**d**) EDS mapping of (**a**,**c**), respectively.

Figure 13a,b show an increase in the size of MC, M_7_C_3_ and M_2_C after tempering at 700 °C. The most significant change in size is observed for M_2_C, which noticeably increases in both length and width, transforming from a needle-like shape at 600 °C to a short rod-like shape. Moreover, after tempering at 700 °C, the characteristics of the high-dislocation density martensite matrix are significantly weakened, and the martensite lath boundaries become less distinct. As shown in Figure 13c,d, dumbbell-like composite carbides are observed, which are similar to the carbides shown in Figure 11d,e. Located at the center of the composite carbides, the MC carbides retained after quenching may provide a carbon source for the nucleation and growth of M_7_C_3_ carbides during tempering at 600–700 °C.

**Figure 14:**
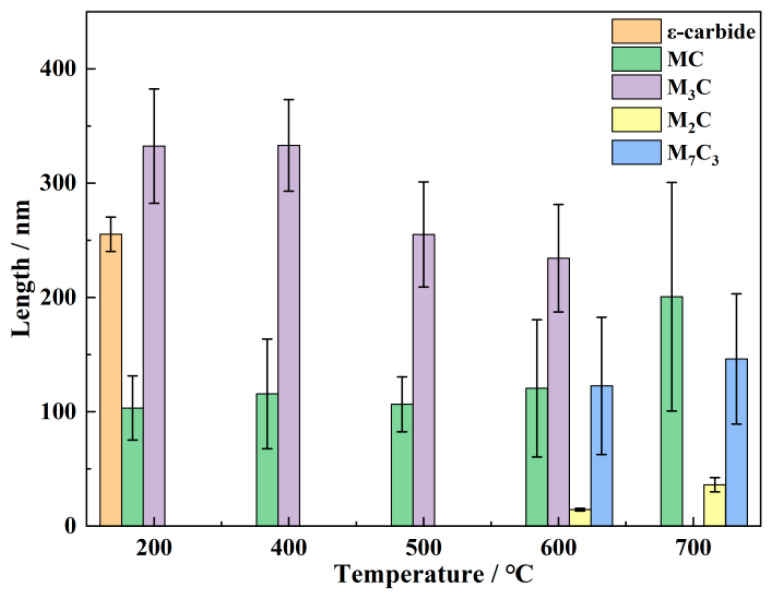
The sizes of various carbides at different tempering temperatures.

Figure 14 presents the sizes of various carbides at different tempering temperatures, as statistically analyzed using Image Pro 7.0 Plus. In this analysis, the sizes of the ε-carbides and M_3_C carbides are measured by their length, while the sizes of the MC, M_2_C and M_7_C_3_ carbides are determined by their equivalent diameter. Due to the fact that larger carbides are prone to falling off during electrolytic double-jet polishing, the size statistics of carbides mainly rely on carbon replicas. The sizes of the MC carbides exhibit no significant changes within the temperature range of 200 °C to 600 °C, but at 700 °C, the average size increases by approximately 66%. MC carbides, remaining undissolved during the quenching process, are considered to be a relatively stable type of carbide. They contribute to precipitation strengthening, but since significant coarsening only occurs at 700 °C, its impact on the changes in strength and toughness is relatively minor. The ε-carbides precipitated at 200 °C provide a certain level of strengthening effect. Disappearing at 400 °C, the ε-carbides are verified to have poor thermal stability at elevated temperatures. Hence, the contribution of ε-carbides to high-temperature strength is negligible. Residual M_3_C carbides from the quenched state are retained and maintain their original state up to 500 °C. However, upon reaching 500 °C, a finer precipitation of M_3_C occurs, which has relatively less contribution to the yield strength and high-temperature strength, as shown in Figure 9. When temperatures reach 600 °C and above, M_3_C carbides begin to decompose and transform into M_7_C_3_ carbides, particularly at 700 °C, where M_3_C carbides completely disappear. However, the formation of larger M_7_C_3_ carbides provides limited strengthening due to their size. In addition, a portion of M_3_C has transformed into alloy cementites at 600 °C. Nano-sized M_2_C carbides are first observed at 600 °C, with their average size being less than one-tenth of that of MC and M_7_C_3_ carbides. Finer M_2_C carbides are known to interact with dislocations effectively, providing a significant strengthening effect. After tempering at 700 °C for 2 h, M_2_C carbides grow to only about 36 ± 6 nm, which is significantly smaller than the MC and M_7_C_3_ carbides. In the high-temperature tensile test at 700 °C, the M_2_C carbides formed after tempering at 600 °C still maintain their nanoscale size, contributing to higher high-temperature strength.

### 3.4. Fracture Morphology

**Figure 15:**
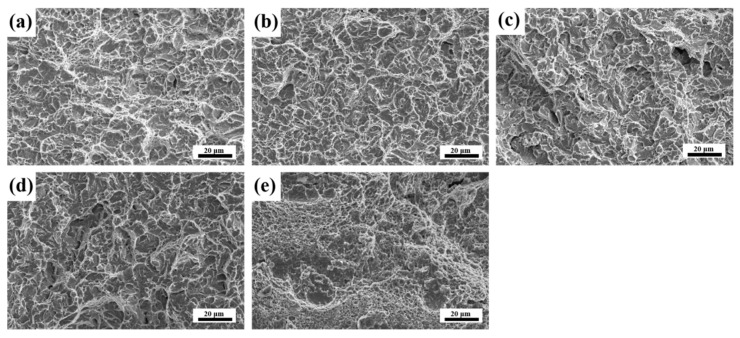
SEM fracture graphs of impact samples tempered at (**a**) 200 °C, (**b**) 400 °C, (**c**) 500 °C, (**d**) 600 °C, (**e**) 700 °C.

Figure 15 depicts the microfracture morphologies at different tempering temperatures. Figure 15a–d show steels tempered between 200 °C and 600 °C exhibit typical quasi-cleavage fracture surface morphologies at −40 °C. Nevertheless, the size and distribution of cleavage facets and dimples within the quasi-cleavage fracture morphologies exhibit variation. Microstructural characteristics and carbide distribution critically govern fracture morphology evolution [39]. Concurrently, dimple formation correlates closely with carbide geometric parameters such as aspect ratio and size. Specimens tempered at 200 degrees (Figure 15a) exhibit enlarged dimples promoted by ε-carbide precipitation. This increases the energy of crack propagation. Through the embrittlement mechanism termed tempered martensite embrittlement, the M_3_C exerts a detrimental effect on impact toughness and ductility [40]. The pronounced hardness gradient at carbide/matrix interfaces induces preferential plastic deformation initiation in the relatively softer matrix phase during impact loading conditions. The resultant stress intensification near hard carbide particles induces localized stress concentrations exceeding critical thresholds, thereby initiating crack nucleation at carbide/matrix interfaces [41,42,43]. In consequence, specimens tempered within 400–500 °C tend to develop quasi-cleavage fracture characteristics (Figure 15b,c), indicative of tempered martensite embrittlement mechanisms. At 600 °C, a turning point in toughness occurs, where a substantial amount of nanoscale M_2_C carbides begin to precipitate in the grain interior. At 600 °C, the quasi-cleavage facets are smaller, and there are more dimples, as shown in Figure 15c,d. The M_2_C carbides can interact with the dislocations, providing a significant level of strength. In addition, nanoscale M_2_C carbides are less likely to form dislocation pile-up sites, thereby making them less prone to generating crack initiation sites [44]. When tempered at 700 °C, the microfracture morphology (Figure 15e) is predominantly composed of dimples. The matrix undergoes substantial dislocation annihilation during 700 °C tempering, accompanied by extensive precipitation, the disappearance of M_3_C and the coarsening of carbides. These factors contribute to the significant increase in toughness.

### 3.5. High-Temperature Strengthening Mechanism

**Figure 16:**
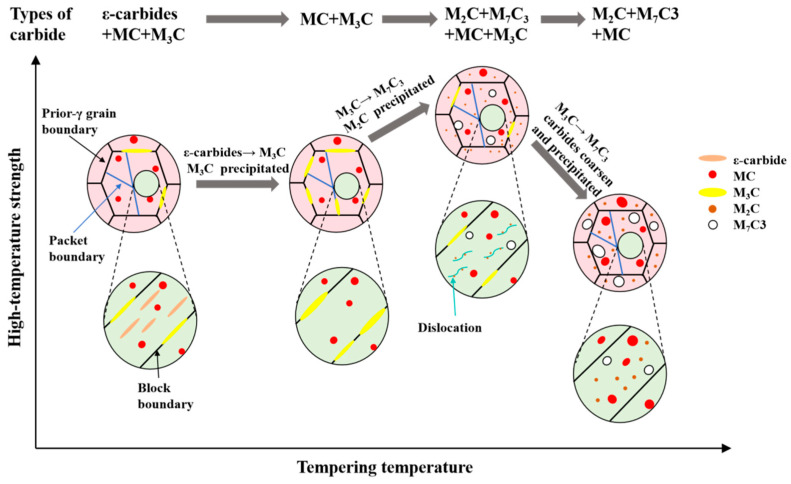
The schematic diagram of the relationship between tempering temperature, carbide evolution and high-temperature strength.

Figure 16 illustrates the pattern of how the evolution of carbides at different tempering temperatures affected high-temperature strength in the steel employed in this work. During the low-temperature tempering stage (200 °C), metastable sub-micron ε-carbides precipitate along the martensitic laths, accompanied by some residual MC and M_3_C carbides from quenching. These carbides collectively contribute to the strengthening effect at high temperatures. However, ε-carbides are prone to transforming into M_3_C at higher temperatures, and the larger size of M_3_C results in a weaker strengthening effect. As observed between 400 and 500 °C, where M_3_C and MC are the predominant carbides, there is little enhancement in high-temperature strength, confirming that M_3_C contributes minimally to high-temperature strength at 700 °C. However, when the tempering temperature reaches 600 °C, a notable difference is observed as massive nanoscale M_2_C precipitates form in the matrix. M_2_C maintains its nanoscale dimensions even under high-temperature conditions, as demonstrated by the tempering state at 700 °C. Therefore, the nanoscale M_2_C with high thermal stability continues to provide a substantial strengthening effect under high-temperature tensile conditions. When the tempering temperature is further increased to 700 °C, various carbides coarsen, particularly the decomposition of M_3_C into coarser M_7_C_3_. A significant amount of alloying elements precipitate out of the matrix in the form of carbides, leading to a substantial reduction in the solid solution strengthening of the martensitic matrix. The combined effects of carbide coarsening and matrix weakening lead to a reduction in high-temperature strength. The evolution of carbides is a significant factor affecting the high-temperature strength of steel, not only reflected in the increment of yield strength but also directly influencing the high-temperature strengthening effect.

## 4. Conclusions

1. Oil-cooling after austenitization at 980 °C produces lath martensite. During the tempering process, ε-carbide is the first carbide to precipitate at 200 °C, and transforms into M_3_C quickly as the temperature rises. Massive precipitation of nanoscale M_2_C and transformation from M_3_C to M_7_C_3_ occur at 600 °C. At 700 °C, accompanying the decomposition of the substructure, a great amount of carbides precipitate and coarsen. The sequential carbide transitions occurring during tempering develop in this order: ε-carbide + MC + M_3_C → MC + M_3_C → MC + M_2_C + M_7_C_3_.
2. M_3_C tends to nucleate and grow at grain boundaries and phase interfaces, and the rod-shaped cementite can be detrimental to toughness, leading to a reduction in toughness from 29.7 ± 1.9 J to 22.4 ± 2.1 J at a tempering temperature of 400 °C. In contrast, fine M_2_C is less likely to cause stress concentration, which results in improved toughness, increasing KV_2_ (−40 °C) from 26.2±0.5 J to 34 ± 1.4 J upon tempering at 600 °C.
3. At high temperatures such as 700 °C, the contribution of carbides to strength is of great significance in steel. Metastable ε-carbides and relatively coarser M_3_C carbides are not sufficient to provide high high-temperature strength. Nanoscale M_2_C with high thermal stability provided both the highest yield strength at room temperature and high-temperature strength, 1340 ± 8 MPa and 421 ± 4 MPa, respectively.

## Figures and Tables

**Table 1:** Summary of experimental matrix and characterization methods.

Tempering Temperature/°C	Mechanical Testing	Microstructural Analysis
200	Room-temperature tensile test + 700 °C high-temperature tensile test + −40 °C Charpy impact test	SEM: microstructure and impact fracture morphology TEM: microstructure EBSD
400	Room-temperature tensile test + 700 °C high-temperature tensile test + −40 °C Charpy impact test	SEM: microstructure and impact fracture morphology TEM: microstructure and carbon extraction replica EBSD
500	Room-temperature tensile test + 700 °C high-temperature tensile test + −40 °C Charpy impact test	SEM: microstructure and impact fracture morphology TEM: microstructure and carbon extraction replica EBSD
600	Room-temperature tensile test + 700 °C high-temperature tensile test + −40 °C Charpy impact test	SEM: microstructure and impact fracture morphology TEM: microstructure and carbon extraction replica EBSD
700	Room-temperature tensile test + 700 °C high-temperature tensile test + −40 °C Charpy impact test	SEM: microstructure and impact fracture morphology TEM: microstructure and carbon extraction replica EBSD

## Data Availability

The original contributions presented in this study are included in the article. Further inquiries can be directed to the corresponding authors.

## Appendix A

Figure A1 shows the SEM (scanning electron microscopy) microstructure of the as-quenched steel and the positions scanned by EDS (energy-dispersive spectroscopy) point scanning. The EDS analysis results indicate that the spherical carbides (A and B) are primarily composed of the alloying elements C, V, Cr and Mo, with an atomic ratio of C to V close to 1:1. This indicates that the carbides are of the MC type, where M represents vanadium (V).

The grain boundary and phase maps of steels tempered at different temperatures are shown in Figure A2. Blue represents M_3_C carbides, red indicates high-angle grain boundaries (>15°), gray denotes low-angle grain boundaries (2–15°), and white signifies the body-centered cubic (BCC) martensitic matrix. From the position indicated by the black dashed line, it can be observed that M_3_C is predominantly distributed along high-angle grain boundaries, whereas within the laths, M_3_C is virtually unobservable. This observation indicates that M_3_C precipitates primarily at the prior austenite grain boundaries and phase interfaces.

Figure A3 illustrates the phase distribution of M_3_C and the BCC matrix at various tempering temperatures. At 500 °C, the amount of M_3_C (cementite) is relatively high, reaching 10%, indicating that M_3_C is the predominant precipitate phase at 500 °C. Furthermore, as the tempering temperature increases to 600 °C and further to 700 °C, the contents of M_3_C decrease gradually, being 5.6% and 3%, respectively.
